# Late diagnosis of celiac disease in an asymptomatic infant with growth failure

**DOI:** 10.1186/1824-7288-40-4

**Published:** 2014-01-15

**Authors:** Mauro Bozzola, Elena Bozzola, Sara Pagani, Amelia Mascolo, Rossella Porto, Cristina Meazza

**Affiliations:** 1 Internal Medicine and Therapeutics Department, University of Pavia, Fondazione IRCCS San Matteo, Pavia, Italy; 2 Pediatric and Infectious Disease Unit, Department of Pediatrics, Bambino Gesù Children's Hospital, Rome, Italy

**Keywords:** Celiac disease, HLA, Malnourishment

## Abstract

The clinical spectrum for celiac disease (CD) is broad and includes cases with either typical (intestinal) or atypical (extraintestinal) features, often making the diagnosis of CD very difficult.

We describe the case of a girl presenting with stunted growth and malnourishment. She was evaluated at 14 months for decreased growth rate without any signs of gastrointestinal, renal or endocrine disorders. She was evaluated for CD, but resulted negative for anti-tTG antibodies.

At the age of 4.1 years, she exhibited basal dental enamel hypoplasia, iron deficiency anaemia despite repeated iron supplementation, with persistent reduced height (-2.79 SDS), BMI (-0.76 SDS), growth velocity (-1.79 SDS) and delayed bone age (1.5 year). The CD screening was repeated and very high anti-tTG-IgA (128 IU/ml, normal values < 7 IU/ml) and anti-tTG-IgG (77 IU/ml, normal values < 7 IU/ml) values were found. HLA genotyping revealed an HLA DQ2 haplotype. A duodenal biopsy revealed severe villous atrophy with crypt hyperplasia and increased intraepithelial lymphocytes (> 40 IELs/100 epithelial cells) confirming the diagnosis of CD. A gluten-free diet was started and after only four months, her growth velocity increased from 4.83 cm/year (-1.79 SDS) to 6.53 cm/year (-0.15 SDS).

In conclusion, we report the development of a positive serology for CD in an asymptomatic child with growth retardation, who previously was investigated for CD and resulted negative. Therefore, when faced with retarded growth in young patients, after excluding other malabsorption conditions and even when CD serological markers are negative, the paediatric endocrinologist should request HLA genotyping, before the intestinal biopsy, in order to check for the presence of risk alleles.

## Background

Celiac disease (CD) is an immune-mediated disease which is triggered by the ingestion of gliadin and other prolamines which are toxic in genetically susceptible subjects. The genetic risk factors for CD have been well characterized. In fact, more than 90% of patients share the major histocompatibility complex II class human leukocyte antigen (HLA)-DQ2 haplotype and most of the remaining subjects carry HLA-DQ8 [1,2]. Subjects negative for both HLA-DQ2 and -DQ8 are very unlikely to suffer from CD. Therefore, HLA genotyping is an important as exam for first degree relatives or as a supporting test when biopsy is excluded due to its invasive nature in symptomatic subjects with high antibody levels [3].

Occasionally, the diagnosis of CD can be difficult in patients in whom serological markers are absent. As suggested by the new ESPGHAN guidelines, if there is a strong suspicion of CD, despite a negative serology, a small bowel biopsy has to be performed [3].

Here, we describe an asymptomatic child with stunted growth and negative serology, in whom CD was not diagnosed at the first evaluation, but later during the follow-up when she became seropositive for CD.

## Case presentation

A 4.1-year-old girl was referred to our department for failure to thrive and anorexia. She was born at term after an uneventful pregnancy with a weight of 3,230 g, a length of 52 cm and head circumference of 34.5 cm. The perinatal period was normal and her Apgar score was 9 at 5 min. She received formula from birth. Gluten was included for the first time at the age of seven months, without any adverse gastrointestinal effects. The target height of the girl was 163 cm (0.13 SDS). Both parents are healthy and unrelated, and had normal development during puberty.

**Figure 1:**
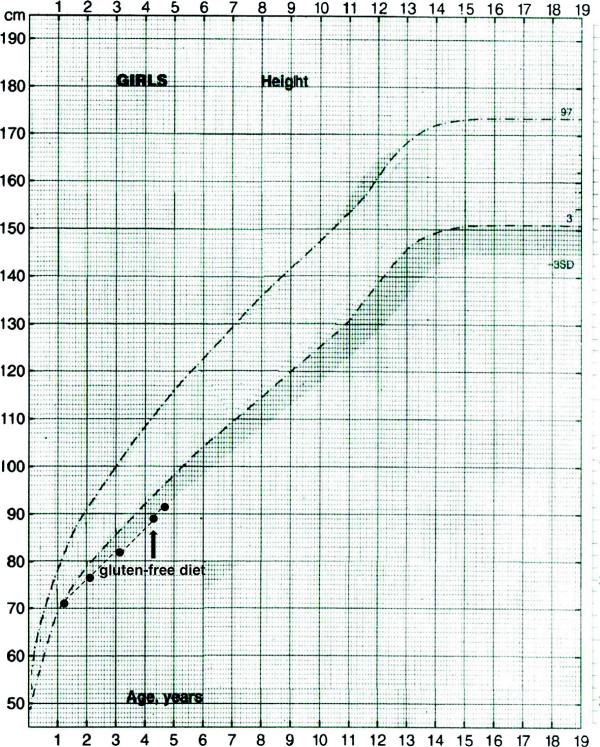
**Height curve of the subject.** The arrow indicates the start of the gluten-free diet.

From the age of six months, she showed a progressive reduction in growth rate, both for weight and height, and at the age of 14 months, she was evaluated at another centre and showed short stature (cm 69.9, -2.19 standard score deviation, SDS) (Figure 1) with adequate weight (7,520 Kg, body mass index (BMI) -0.45 SDS), free thyroxin (FT4) and thyroid-stimulating hormone (TSH) were within the normal range. Furthermore, serum anti-transglutaminase (anti-tTG) antibodies were negative (IgA-tTG 1.7 IU/ml and IgG-tTG 7.2 IU/ml; normal values <7 IU/ml) with normal for age circulating IgA values (38 mg/100 ml; normal values: 37–257 ng/ml). She exhibited iron deficiency anaemia, but the sweat test (Na^+^ 16 mEq/l and Cl^-^ 13 mEq/l; normal values: Na^+^ and Cl^-^ <40 mEq/l) and peripheral T and B cell assessment resulted normal. No abnormalities of the kidneys were detected by ultrasound and no gastrointestinal disorders were reported.

At the moment of our evaluation, at the age of 4.1 years, physical examination revealed only basal dental enamel hypoplasia. She showed a height of 89.5 cm (-2.79 SDS) (Figure 1), a weight of 12,5 Kg (BMI -0.76 SDS), a delayed bone age of 1.5 years, and a growth velocity during the last year of 4.83 cm/year (-1.79 SDS). Iron deficiency anaemia persisted despite cyclically repeated iron supplementation. The screening for CD was repeated and very high IgA -tTG (128 IU/ml) and IgG-tTG (77 IU/ml) values were observed. These data were highly suggestive of CD. HLA genotyping revealed DQA1*02:01, 05:05 and DQB1 *02:02, 03:01 haplotypes (i.e. HLA DQ2 haplotype). A duodenal biopsy revealed severe villous atrophy (Marsh 3c) with crypt hyperplasia and increased intraepithelial lymphocytes (> 40 IELs/100 epithelial cells) confirming the diagnosis of CD. A gluten-free diet was started and after only four months her growth velocity increased from 4.83 cm/year (-1.79 SDS) to 6.53 cm/year (-0.15 SDS).

HLA genotyping in first-degree relatives revealed DQA1*05:01, 05:05 and DQB1*02:01, 03:01 in her older brother, who had a negative serology for CD. Therefore, his diet includes gluten and he repeats CD serology testing every three years.

## Discussion

Celiac disease is not simply a gastrointestinal condition, but an immune-mediated systemic disorder, strongly dependent on HLA-DQ2 and DQ8 haplotypes. At present, diagnosing CD is often difficult, due to the reduced prevalence of the classical form with gastrointestinal symptoms and the increased prevalence of atypical forms [4]. Clinical suspicion should be supported by positive serological tests and diagnosis requires typical histopathological findings with a biopsy of the small intestine, especially when the patient is asymptomatic [3]. It is important to stress that early diagnosis of CD with prompt initiation of a gluten-free diet can decrease the potential risk of both adenocarcinoma and lymphoma, which occurs more frequently in untreated celiac patients than in the general population [5].

This report emphasizes the difficulty of diagnosing CD in asymptomatic patients with a negative serology who exhibit only stunted growth. In fact, when this patient was first evaluated, she resulted negative for anti-tTG, her only symptom was progressive growth failure.

The negative serology observed in our patient at the 14th month of life could be related to the lower sensitivity of anti-tTG antibodies in children younger than two years, as recently described by Maglio et al. [6]. We did not measure antibodies against native gliadin (AGA) which is indicated in children up to two years old [7]. However, a recent study suggested that IgA-anti-tTG results are more reliable than AGA, also in younger children [8].

Then, interestingly after about a three year-follow-up, high serum levels of anti-tTG unexpectedly appeared in the absence of typical CD gastrointestinal features. Furthermore, the HLA genotyping showed one haplotype linked to CD susceptibility. Interestingly, a different haplotype linked to CD susceptibility was found in her asymptomatic older brother who currently exhibits a negative serology. We may speculate that this difference is due to a different gene dose effect of the allele that contributes to the development of CD. In fact, in several studies the risk of CD has been found to be significantly greater in subjects homozygous for the DQB1*0201 allele than in subjects heterozygous for the same allele [9,10]. However, both subjects have DQ2 haplotype in the heterozygous state (although with different risk alleles). On the other hand, conflicting data have been published on the gene dose effect and the number of risk alleles on the age of CD diagnosis and severity of symptoms [11-13].

It is well known that the sensitivity and specificity of HLA genotyping are sufficiently high to exclude CD only when all risk alleles including DQ2 and DQ8 are absent. The ESPGHAN guidelines for the diagnosis and management of CD in children suggest HLA genotyping in asymptomatic children with associated conditions and negative serology. When HLA DQ2/DQ8 is positive, surveillance should continue and serology for CD should be repeated every three years [3]. On the contrary, when HLA DQ2/DQ8 is negative, the development of CD is highly unlikely and regular antibody screening may be discontinued [3]. HLA genotyping is also useful in the risk evaluation for CD in 1st degree relatives of patients with CD [14].

In conclusion, we report the development of positive serology for CD in an asymptomatic child with growth retardation, who previously was evaluated for CD and had resulted negative. Therefore, when faced with retarded growth in young patients, the paediatric endocrinologist should always maintain a high index of suspicion for CD, even if serological markers are negative. Furthermore, in some selected cases with failure to thrive and negative serology for CD, after the exclusion of other malabsorption conditions, the paediatrician could request HLA genotyping, before the intestinal biopsy, in order to check for the presence of risk alleles.

## Consent section

Written informed consent was obtained from the patient’s parents for publication of this Case report and any accompanying images. A copy of the written consent is available for review by the Editor-in-Chief of this journal.

## Abbreviations

CD: Celiac disease; HLA: Human leukocyte antigen; SDS: Standard deviation score; BMI: Body mass index; Anti-tTG: Anti-tissue transglutaminase; Ig: Immunoglobulin; FT4: Free thyroxin; TSH: Thyroid stimulating hormone; AGA: Anti-gliadin antibodies.

## Competing interests

The authors declare that they have no competing interests.

## Authors’ contributions

MB collected the patient data and drafted the manuscript. EB helped to draft the manuscript. SP helped to collect the data and draft the manuscript. RP and AM participated in the diagnosis and critically revised the manuscript. CM contributed to the interpretation of the data and helped to draft the manuscript. All the authors approved the final version of the manuscript.
